# Efficient BST2 antagonism by Vpu is critical for early HIV-1 dissemination in humanized mice

**DOI:** 10.1186/1742-4690-10-128

**Published:** 2013-11-06

**Authors:** Vibhuti P Dave, Fadi Hajjar, Mame Massar Dieng, Élie Haddad, Éric A Cohen

**Affiliations:** 1 Laboratory of Human Retrovirology, Institut de Recherches Cliniques de Montréal (IRCM), 110 Pine avenue west, Montreal, QC H2W 1R7, Canada; 2 CHU Sainte-Justine Research Center, 3175 chemin de la Côte-Sainte-Catherine, Montreal, QC H3T 1C5, Canada; 3 Department of Paediatrics, Université de Montréal, 2900 Boul. Édouard- Montpetit, Montreal, QC H3T 1J4, Canada; 4 Departments of Microbiology, Infectiology and Immunology, Université de Montréal, 2900 Boul. Édouard- Montpetit, Montreal, QC H3T 1J4, Canada

**Keywords:** Vpu, BST2, HIV-1 release, Humanized mice, Viral dissemination

## Abstract

**Background:**

Vpu is a multifunctional accessory protein that enhances the release of HIV-1 by counteracting the entrapment of nascent virions on infected cell surface mediated by BST2/Tetherin. Vpu-mediated BST2 antagonism involves physical association with BST2 and subsequent mislocalization of the restriction factor to intracellular compartments followed by SCF(β-TrCP) E3 ligase-dependent lysosomal degradation. Apart from BST2 antagonism, Vpu also induces down regulation of several immune molecules, including CD4 and SLAMF6/NTB-A, to evade host immune responses and promote viral dissemination. However, it should be noted that the multiple functions of Vpu have been studied in cell-based assays, and thus it remains unclear how Vpu influences the dynamic of HIV-1 infection in *in vivo* conditions.

**Results:**

Using a humanized mouse model of acute infection as well as CCR5-tropic HIV-1 that lack Vpu or encode WT Vpu or Vpu with mutations in the β-TrCP binding domain, we provide evidence that Vpu-mediated BST2 antagonism plays a crucial role in establishing early plasma viremia and viral dissemination. Interestingly, we also find that efficient HIV-1 release and dissemination are directly related to functional strength of Vpu in antagonizing BST2. Thus, reduced antagonism of BST2 due to β-TrCP binding domain mutations results in decreased plasma viremia and frequency of infected T cells, highlighting the importance of Vpu-mediated β-TrCP-dependent BST-2 degradation for optimal initial viral propagation.

**Conclusions:**

Overall, our findings suggest that BST2 antagonism by Vpu is critical for efficient early viral expansion and dissemination during acute infection and as such is likely to confer HIV-1 increased transmission fitness.

## Background

Studies over the last decade have provided ample evidence to suggest that host cells deploy multiple mechanisms to counter human immunodeficiency virus (HIV) infection. These intrinsic/innate antiviral host cell responses are mediated by an array of host restriction factors such as APOBEC3F/G, SamHD1, and BST2 (also called Tetherin, CD317, HM1.24). However, HIV has evolved to encode accessory proteins such as Vif, Vpx and Vpu to antagonize these host restriction factors, thus permitting efficient viral replication and evasion of innate immune responses [1,2]. One extensively studied host restriction factor is BST2, a protein that is broadly induced by type 1 interferon (IFN) in various cell types, including CD4^+^ T cells and macrophages, suggesting important roles for this factor in antiviral innate responses [3-5]. BST2 is a type II membrane protein that contains two membrane anchors imparting a unique topology to the protein. It consists of a short N-terminal cytosolic tail, a transmembrane domain (TMD), an extracellular portion that is predominantly helical and a glycophosphatidyl-inositol (GPI)-linked membrane anchor present at the C-terminus. This distinctive configuration permits BST2 to insert one membrane anchor into budding HIV-1 particles and thus inhibit the release of virions through tethering of nascent viral particles to the plasma membrane (PM) [4-7]. While it is generally agreed that BST2 impairs the release of cell-free HIV-1 particles, it is a subject of debate whether the BST2 mediated restriction affects viral cell-to-cell transmission, the most efficient mode of HIV-1 dissemination [8,9]. Nevertheless, study of mice deficient for BST2 or encoding single nucleotide polymorphism in BST2 have underlined the importance of this effector of the IFN antiviral response in restricting retrovirus replication and disease progression *in vivo*[10,11].

To counter the ability of BST2 to inhibit virus release from infected cells, HIV-1 encodes Vpu, which was the first BST2 antagonist to be identified [4,5]. Vpu is a type I membrane protein containing a TMD and a carboxy-terminal cytoplasmic tail domain (CTD). The past few years have seen extensive characterization of Vpu-BST2 interactions and these studies have shown that the TMD of Vpu and BST2 are crucial for direct physical interactions between the two proteins and indeed govern the species specific basis of BST2 antagonism [12-14]. Importantly, Vpu interaction with BST2 results in mislocalization of the restriction factor from the PM to endosomal and perinuclear compartments, namely the TGN, thus leading to an overall reduction of BST2 levels at the cell surface [15-18]. Thus, mutating Vpu at specific positions in the TMD resulted in failure to down regulate BST2 from the cell surface and inefficient HIV-1 release [12,14].

Apart from the TMD, the CTD of Vpu is also involved in BST2 antagonism. This region of Vpu consists of two alpha helices separated by a hinge region containing two phosphorylatable serine residues, S52 and S56, that are required to recruit β-TrCP, a substrate recognition module of the Skp1/Cullin/Fbox (SCF^β-TrCP^) E3 ubiquitin ligase complex [19]. Increasing evidence suggests that SCF^β-TrCP^-dependent ubiquitination of the restriction factor contributes to BST2 antagonism by targeting cell surface BST2 as well as de novo synthesized BST2 for degradation in the lysosomes [15,20-23]. It should be noted that preventing Vpu-mediated β-TrCP dependent degradation of BST2 does not completely abolish BST2 antagonism in several cellular models [16,17,24-27], indicating that Vpu can inhibit BST2 function independently of degradation. Similarly, putative sorting motifs present in the CTD of Vpu also play critical roles in targeting BST2-Vpu complex to intracellular compartments where BST2 is precluded from the area of viral assembly at PM [25,28].

Vpu also performs several other functions with implications for immune response as well as viral release and infectivity. Thus, Vpu induces the ubiquitination and proteasomal degradation of newly synthesized CD4 molecules that form a complex with the envelop precursor gp160 (Env) in the endoplasmic reticulum (ER) via a mechanism that strictly depends on β-TrCP recruitment [19,29-31]. It should be noted that HIV-1 down regulates and degrades CD4 not only through Vpu, but also in concert with Env and Nef viral proteins. Although the biological relevance of HIV-1-mediated CD4 down regulation is still unclear, it could enhance viral infectivity by alleviating CD4 sequestration of Env, a condition that impedes efficient trafficking of Env towards viral assembly sites at the PM, and/or prevent superinfection [32-34]. Recent data also suggest that Vpu down regulates cell-surface immune molecules such as the co-activating NK receptor NK, T-cell, B cell antigen (NTB-A) (also termed CD352 or SLAMF6) [35] as well as, to a lesser extent, PVR (also termed CD155 or Necl-5), the ligand of the activating NK cell receptor DNAM-1 [36], to evade recognition and killing of infected T cells by NK cells.

The aforementioned multiple functions of Vpu have been largely studied employing permissive and non permissive cell lines or primary HIV target cells such as CD4^+^ T cells and macrophages. However, recent technological advances have made it possible to employ humanized mouse (hu-mice) model for studies of HIV-1 infection under *in vivo* conditions. Indeed, a recent study demonstrated that hu-mice infected with Vpu-sufficient but not -deficient HIV-1 exhibited an initial burst phase of viral propagation that correlated with a down regulation of BST2 and CD4 in infected CD4^+^ T cells [37]. However, the consequence of Vpu-mediated target protein trafficking alteration and/or degradation under *in vivo* condition remains to be dissected. For instance, it is not clear if impairing Vpu-mediated ubiquitination and degradation of BST2 and CD4 (via recruitment of β-TrCP) has the same effect on HIV-1 replication and dissemination.

In this study, we used the humanized NOD-scid IL2Rγ^null^ (NSG) mouse model of acute HIV infection as well as CCR5-tropic HIV-1 virus that lack Vpu or encode WT Vpu or Vpu with mutations in the β-TrCP-binding site to assess the role of Vpu-mediated BST2 antagonism in establishing efficient plasma viremia and viral dissemination in lymphoid tissues during infection *in vivo.*

## Results

### Impact of Vpu-deficiency on HIV-1 replication and propagation in hu-mice

**Figure 1:**
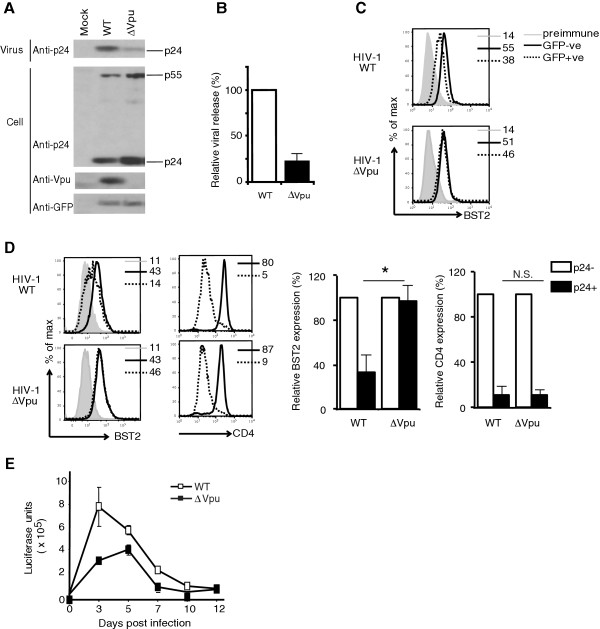
**Characterization of CCR5-tropic HIV-1-WT and HIV-1-**∆**Vpu proviral DNA and viruses.** HeLa cells were transfected with WT or Vpu-deficient pNL4.3-Ada-GFP proviral DNA. Transfected cells and virus-containing supernatants were analyzed by western blot using the indicated antibodies **(A)**. Relative virus particle release efficiency. The particle release efficiency of HIV-1-WT was set at 100% (average of 3 independent experiments) **(B)**. An aliquot of transfected cells was used for analysis of BST2 levels at the surface of GFP-positive (HIV-expressing cells; dashed line) and -negative (non-transfected cells; continuous line) cells by flow cytometry (representative of n = 3). Gray filled histogram represents preimmune staining with normal rabbit serum **(C)**. Primary CD4^+^ T cells were isolated from healthy donors and activated with PHA and IL-2. Activated T cells were infected with HIV-1-WT or HIV-1-∆Vpu at an MOI of 1. At different time points post infection, an aliquot of culture was collected for flow cytometry analysis of CD4 and BST2 expression at the surface of GFP-positive (infected; dashed line) or -negative (non infected; continuous line) cells. Data is shown for cells collected 3 days post infection (dpi). Relative BST2 and CD4 levels at 3-dpi on p24^-^ (open bar) and p24^+^ (filled bar) cells are shown (MFI on p24-negative = 100%; n = 2, * p ≤ 0.05, N.S.: not significant). **(D)**. Infectious virus production in supernatant was determined by assessing the levels of luciferase activity (in duplicate) following infection of HeLaTZM-bl reporter cells **(E)**. Data are representative of 3 independent experiments. Error bars represent standard deviations (SD).

A group of NSG mice reconstituted with human cord-blood-derived CD34^+^ stem cells (hu-mice) were infected with CCR5-tropic HIV-1 (pNL4.3-Ada-GFP) expressing Vpu (HIV-1-WT) or lacking Vpu (HIV-1-∆Vpu). It is important to note that the Vpu protein encoded by pNL4.3-Ada-GFP originates from the Ada strain and not the prototypical pNL4.3 variants. Transfection of HeLa cells with WT and ∆Vpu proviral DNA revealed that WT HIV-1 down regulated endogenously expressed surface BST2 in a Vpu-dependent manner and that reduction of BST-2 levels at the surface of HIV-1 producing cells correlated with efficient virus particle release (Figure 1A-C). Furthermore, infection of activated primary human CD4^+^ T cells also showed a requirement of Vpu in BST2 down regulation and efficient production of infectious virus. Lastly, both viruses had the ability to down regulate the CD4 receptor at the cell surface with what appeared a minor but reproducible contribution resulting from Vpu (Figure 1D-E). However, statistical significance could not be achieved.

**Figure 2:**
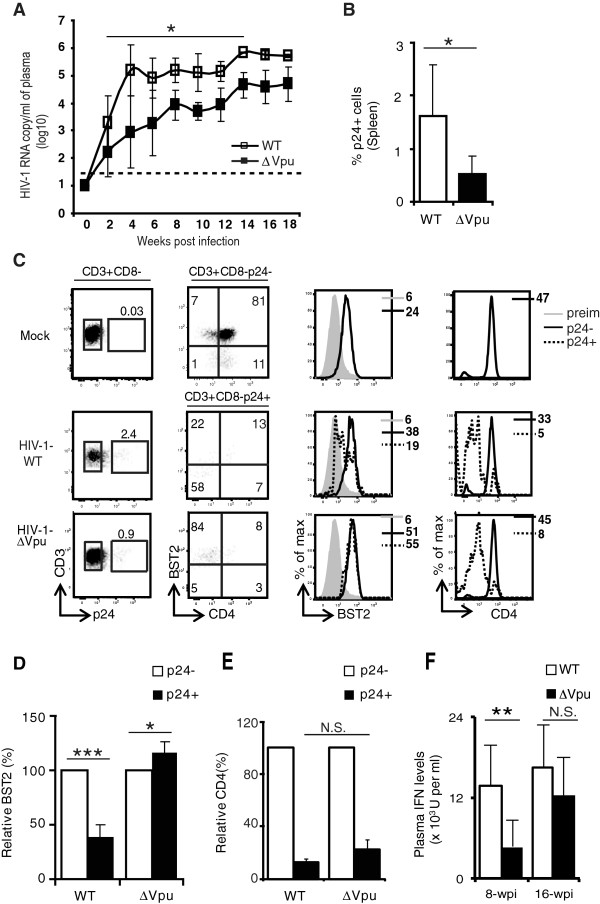
**Impact of Vpu-sufficiency and -deficiency on the dynamic of HIV-1 infection in hu-mice infected with low dose of virus. (A)** Kinetics of plasma viral load was measured by determining RNA copy numbers/ml (log_10_ values) in plasma at different time following infection of hu-mice with low dose of HIV-1-WT or HIV-1-∆Vpu (n = 4 up to 10-wpi and n = 2 after 10-wpi for HIV-1-WT, and n = 4 for HIV-1-∆Vpu at all time points); the horizontal broken line indicates the detection limit of the viral load assay (40 copies/ml). Viral load for mock-infected animals was less than log_10_ value of 2. **(B and C)** T cells in spleen of low dose infected hu-mice were stained at 8 to 10-wpi with a combination of antibodies to identify infected and uninfected CD4^+^ T cells. **(B)** Comparison of average frequency of p24^+^ T cells in spleen of hu-mice infected with HIV-1-WT and HIV-1-∆Vpu (data pooled from 2 independent experiments; n ≥ 5). **(C)** To determine the impact of Vpu on surface BST2 levels, infected (p24^+^CD3^+^CD8^-^) or uninfected (p24^-^CD3^+^CD8^-^) T cells were gated (first column) and BST2 and CD4 expression was analyzed by two-color dot plots (CD4 versus BST2 (second column) as well as by single color histograms (third and fourth columns). Numbers in the first column represent frequency of p24^+^ T cells, and numbers in the upper and bottom left quadrants of dot plots represent BST2^+^CD4^-^ and BST2^-^CD4^-^ frequency in p24^+^ T cells. **(D)** Comparison of relative levels of BST2 on uninfected (p24^-^) and infected (p24^+^) T cells from spleen of hu-mice infected with the indicated HIV-1 virus (MFI on p24^-^ T cells = 100%; n ≥ 5;). **(E)** Bar graph for relative CD4 down regulation on p24^+^ T cells from hu-mice infected with the indicated HIV-1 virus relative to p24^-^ cells (MFI on p24^-^ T cells = 100%). **(F)** IFN levels were determined at 8-wpi and 16-wpi in plasma of hu-mice infected with WT or ∆Vpu HIV-1 (for 8-wpi n = 4 for both groups and for 16-wpi n = 2 for WT and n = 4 for ∆Vpu). Error bars represent SD; *, p ≤ 0.05; **, p = 0.002; ***, p ≤ 0.0005. N.S.: not significant.

To determine the impact of Vpu on viral replication and propagation under *in vivo* conditions, we initially infected hu-mice with low dose (~5,000 TCID_50_) of HIV-1-WT or HIV-1-∆Vpu virus. Hu-mice were bled every alternate week for up to 18 weeks post infection (wpi) for estimation of viral load in plasma and frequency of CD4^+^ T cells in the blood. As shown in Figure 2A, HIV-1-WT-infected hu-mice showed detectable levels of plasma viral load as early as 2-wpi and it increased further at 4-wpi, a level that was maintained up to 18-wpi. In contrast, HIV-1-∆Vpu infected hu-mice showed delayed and reduced plasma viral load kinetics especially at early time points (2–6 wpi) with peak viral load achieved only between 12- and 16-wpi. Thus at 4- and 18-wpi average plasma viral load in HIV-1-WT infected hu-mice was ~150- and ~5-fold more compared to HIV-1-∆Vpu infected animals. Interestingly, the differences in absolute plasma viremia between the two groups of hu-mice became less significant 14-wpi onwards, indicating that over time HIV-1-∆Vpu replication could reach levels similar to those of HIV-1-WT and suggesting that HIV-1 -∆Vpu virus are ultimately able to overcome host cell restrictions. Analysis of peripheral blood T cells showed that the average frequency of p24^+^ T cells in blood from HIV-1-WT infected hu-mice was higher than that from their HIV-1-ΔVpu infected counterparts especially at early time points (4-8wpi); however, statistical significance could not be achieved due to large variations in frequency of p24^+^ T cells in individual hu-mice (Additional file 1: Figure S1A). Detection of infected cells by measurement of virus-encoded GFP could not be used as a substitute as it was less sensitive compared to Gag staining. Moreover, a decrease in CD4^+^ T cell frequency in blood was observed at 12-wpi and later time points in HIV-1-WT infected hu-mice compared to HIV-1-ΔVpu infected hu-mice (Additional file 1: Figure S1B). This fastest rate of CD4^+^ T cell depletion by HIV-1 WT virus most probably reflects the more rapid infection dynamic of these viruses relative to their ΔVpu counterparts.

To study the effect of Vpu on viral dissemination and to determine whether Vpu deficiency affected BST2 and CD4 expression on infected T cells *in vivo*, we sacrificed hu-mice at 8 to 10-wpi for analysis of splenic cells by flow cytometry. Figure 2B and 2C show analysis and frequency of infected (p24^+^) T cells in the CD3^+^CD8^-^ splenic subset of hu-mice infected with WT or Vpu-deficient HIV-1. It should be noted that more than 90% of CD3^+^CD8^-^ cells belong to the CD4 lineage (see top panel in second column of Figure 2C). Spleen of hu-mice infected with HIV-1-WT contained ~ three times more p24^+^ cells than those of hu-mice infected with HIV-1-∆Vpu, indicating that WT virus disseminated more efficiently to lymphoid tissues than HIV-1-∆Vpu (Figure 2B). Flow cytometric study of surface BST2 expression revealed that compared to mock-infected hu-mice, expression of the restriction factor was increased on uninfected CD4^+^ T cells from WT or ∆Vpu HIV-1 infected hu-mice (Figure 2C) very likely because of production of human type I IFN in these mice as shown below (Figure 2F). We also noticed an average of 15-20% BST2 up regulation in infected T cells from HIV-1-∆Vpu infected hu-mice and this was significant compared to BST2 expression on uninfected T cells from the same animals (Figure 2C-D) Importantly, surface BST2 expression was down regulated by 50-75% on p24^+^ splenic T cells from HIV-1-WT but not on those from HIV-1-∆Vpu infected hu-mice (Figure 2C-D). Interestingly, p24^+^ T cells from both groups of infected hu-mice displayed comparable levels of cell-surface CD4 down regulation relative to p24^-^ T cells, consistent with the remaining ability of both viruses to down regulate CD4 through Nef and Env (Figure 2C and 2E). Noticeably, measurement of type I IFN levels in the plasma at different time points revealed a marked difference between WT and ∆Vpu HIV-1 infected hu-mice at early time points (8-wpi) (Figure 2F); however this difference became non significant at later time points (16-wpi), suggesting that the decreased difference in plasma viremia observed over time between the two groups of mice is not due to lower IFN production nor by extension to lower BST2 levels. These findings indicate that Vpu is critical for down regulating BST2 expression *in vivo*, and that this effect most likely correlates with efficient initial viral production and propagation in hu-mice infected with low dose of HIV-1.

### Effect of Vpu on the dynamic of viral infection at supra-physiological dose of HIV-1

**Figure 3:**
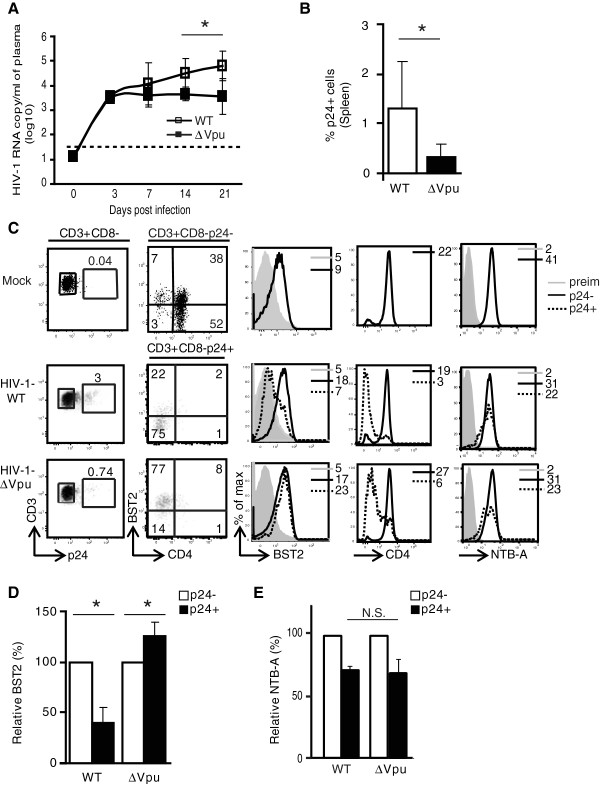
**Infection of hu-mice with supra physiological dose of HIV-1-∆Vpu does not overcome BST2 restriction on early viral propagation.** Hu-mice were infected with an inoculum containing 100-fold more (compared to low dose) infectious HIV-1-WT or HIV-1-∆Vpu and bled at 3, 7, 14 and 21-dpi. **(A)** shows RNA copy number/ml of plasma (log_10_ values) at indicated time points in hu-mice infected with HIV-1-WT or HIV-1 ∆Vpu virus (n = 5). The horizontal broken line depicts the detection limit of the viral load assay as in the Figure 1. **(B)** Frequency of p24^+^ T cells at 21-dpi in spleen of hu-mice infected with HIV-1-WT or HIV-1-∆Vpu (n ≥ 4). **(C)** Impact of Vpu on BST2, CD4 and NTB-A levels on p24^-^ and p24^+^ T cells from individual hu-mouse infected with the indicated HIV-1 virus. **(D)** Comparison of relative BST2 levels on p24^+^ and p24^-^ T cells from spleen of hu-mice inoculated with the indicated HIV-1 virus at 21 dpi (MFI on p24^-^ T cells = 100%; n ≥ 4). **(E)** Bar graph for relative NTB-A down regulation on p24^+^ T cells compared to p24^-^ T cells from hu-mice infected with the indicated HIV-1 virus at 21dpi. Error bars represent SD; *, p ≤ 0.05; N.S.: not significant.

To investigate if exposing hu-mice to a supra-physiological dose of infectious HIV-1-∆Vpu virus could overcome the BST2 restriction on early HIV-1 expansion, we infected a cohort of hu-mice with a 100-fold more virus (~500,000 TCID_50_ of HIV-1-WT or -∆Vpu virus). The infected hu-mice were bled at 3, 7, 14 and 21-days post infection (dpi). Hu-mice challenged with high dose of HIV-1-WT or -∆Vpu showed detectable amounts of virus by 3-dpi. However, compared to HIV-1-∆Vpu infected animals, HIV-1-WT infected animals showed rapid increase in plasma viremia at subsequent time points. In fact, by 14- and 21-dpi there was an ~9- and ~15-fold increase, respectively, in the average amounts of virus in plasma of HIV-1-WT infected animals compared to HIV-1-∆Vpu infected animals (Figure 3A). In agreement with higher plasma viral load, average frequency of p24^+^ T cells at 21-dpi was ~ four-fold higher in spleen of hu-mice infected with HIV-1-WT than those challenged with Vpu-deleted HIV-1 (Figure 3B-C), and the infected T cells from HIV-1-WT but not -∆Vpu challenged hu-mice showed significant down regulation of BST2 (Figure 3C-D). That lower plasma viral load in HIV-1-∆Vpu infected hu-mice was largely due to impaired BST2 down regulation is supported by the fact that Vpu-deficient and proficient virus down regulated CD4 (Figure 3C) or NTB-A (Figure 3C and 3E) to a comparable extent. These data strongly suggest that in the absence of Vpu, infection of hu-mice with high dose of HIV-1 still fails to completely overcome BST2 restriction, thus highlighting the critical barrier that BST2 restriction might represent during acute infection *in vivo*. Collectively, our results suggest that Vpu-mediated BST-2 antagonism is critical for HIV-1 replication and propagation *in vivo*, especially at early times post-infection.

### Contribution of the E3 ubiquitin ligase recruiting domain of Vpu in HIV-1 production and dissemination in hu-mice

**Figure 4:**
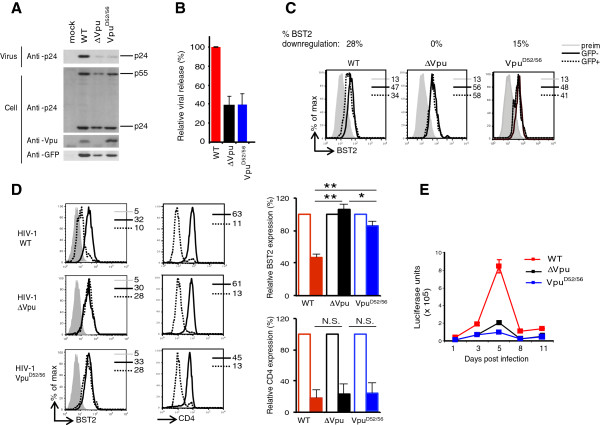
**Characterization of HIV-1 proviral DNA encoding the Vpu S52/56 mutant.** HeLa cells were transfected with HIV-1-WT, HIV-1-∆Vpu or HIV-1-Vpu^D52/56^ proviral DNA (pNL4.3-Ada-GFP backbone). Transfected cells and virus-containing supernatants were analyzed by Western blot using the indicated antibodies **(A)**. Relative viral particle efficiency. The particle release efficiency of HIV-1-WT was set at 100% (average of 3 independent experiments) **(B)**. Analysis of cell-surface BST2 expression by flow cytometry **(C)**. Data are representative of 3 independent experiments. PHA and IL-2 activated primary CD4^+^ T cells were infected with HIV-1-WT, HIV-1-∆Vpu, or HIV-1-Vpu^D52/56^ virus at an MOI of 1. At different time points post infection, cells were collected for flow cytometric analysis of CD4 and BST2 expression **(D)**. Data is shown for cells collected at 3-dpi. Relative BST2 and CD4 levels at 3-dpi on p24^-^ (open bar) and p24^+^ (filled bar) cells are shown (MFI on p24-negative = 100%; n = 3). Infectious virus production was determined in supernatants as described in Figure 1**(E)**. Data are representative of 3 independent experiments. Error bar represents SD; * p ≤ 0.05, ** p ≤ 0.005, N.S.: not significant.

While cell culture studies have provided important insight into the mechanistic basis of Vpu-mediated BST2 antagonism and HIV-1 release, it is not clear how Vpu mutations that impair β-TrCP recruitment and inhibit Vpu-mediated BST2 degradation affect HIV-1 infection *in vivo*. To this end, we mutated the two CTD serine residues at positions 52 and 56 for negatively charged aspartic residues (HIV-1-Vpu^D52/56^) in the context of the pNL4.3-Ada-GFP proviral DNA (HIV-1-WT) since we have previously shown that these substitutions prevented the recruitment of β-TrCP and inhibited BST2 degradation [16]. Transfection of HeLa cells with HIV-1-Vpu^D52/56^ proviral DNA or infection of activated primary human CD4^+^ T cells with HIV-1-Vpu^D52/56^ revealed that the β-TrCP-binding mutant was strongly attenuated in its ability to promote HIV-1 particle release and down regulate surface BST2, yet was able to down regulate CD4 at the cell surface to levels comparable to Vpu-deficient or proficient viruses. Noticeably, in the context of VpuAda, mutation of the β-TrCP-interacting S52/56 motif did not display residual BST2 antagonism in HeLa cells and showed an impairment of virus production, which was comparable to that of Vpu-defective virus in activated CD4^+^ T cells (Figure 4).

**Figure 5:**
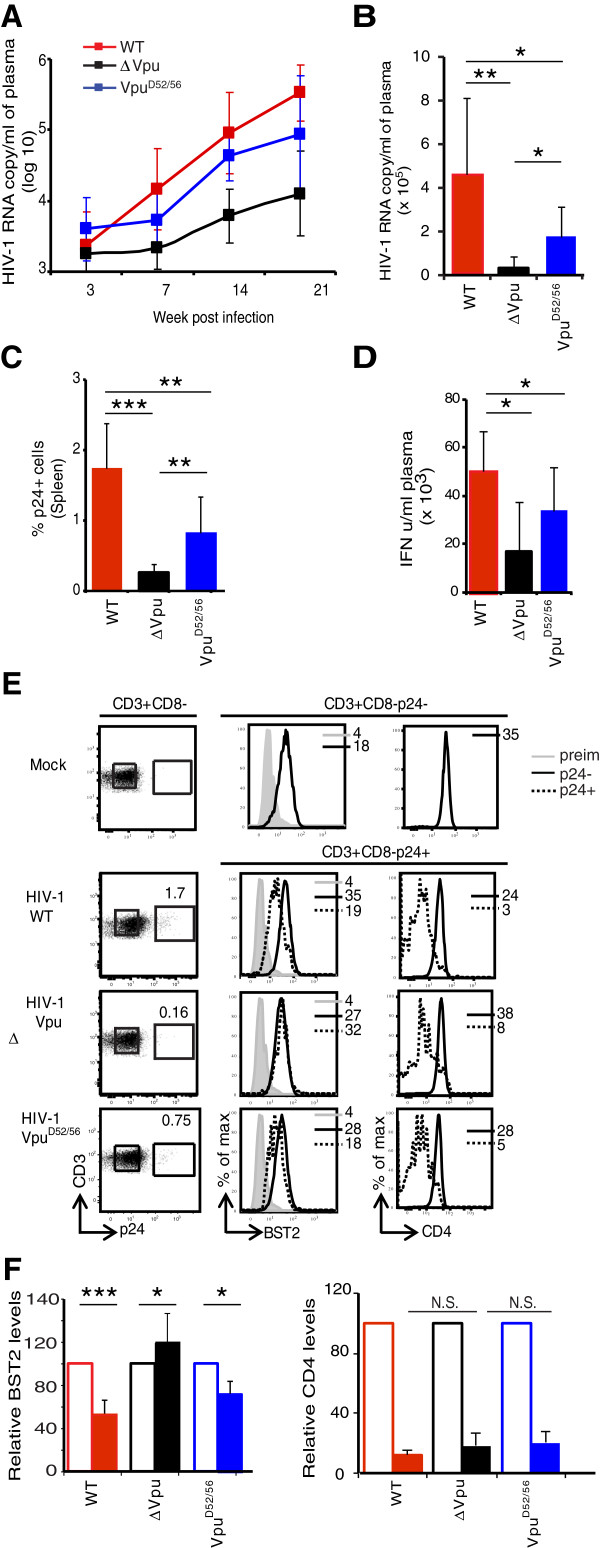
**Effect of Vpu mutated in the β-TrCP-binding domain on the dynamics of HIV infection in hu-mice.** Hu-mice were infected with high dose of HIV-1-WT, HIV-1-∆Vpu, or HIV-1-Vpu^D52/56^ and plasma viral load was determined at different time points. **(A)** shows kinetics of RNA copy number per ml of plasma (log_10_) and **(B)** shows absolute values at 21-dpi in plasma of hu-mice infected with the indicated HIV-1. Please note that x-axis crosses at log_10_ value of 3.0. **(C)** shows comparison of the frequency of p24^+^ T cells in spleen of hu-mice infected with the indicated HIV-1 (n ≥ 7) at 21-dpi. **(D)** shows type I IFN levels at 21-dpi in plasma of hu-mice infected with the indicated HIV-1. **(E)** shows impact of Vpu mutations on BST2 and CD4 levels on p24^+^ and p24^-^ T cells from individual hu-mouse infected with the indicated strain of HIV-1. **(F)** shows comparison of relative BST2 and CD4 levels on p24^+^ (closed bar) and p24^-^ (open bar) T cells from spleen of hu-mice infected with the indicated strain of HIV-1 at 21-dpi (n ≥ 4). BST2 and CD4 MFIs on p24-negative uninfected cells were treated as 100%. Error bars represent SD; *, p ≤ 0.05; **, p ≤ 0.005, ***, p ≤ 0.0005, N.S.: non significant.

To assess the effect of abrogating Vpu binding to β-TrCP on BST2 antagonism and early HIV-1 propagation, we infected hu-mice with high dose (~500,000 TCID_50_) of HIV-1-WT, HIV-1-∆Vpu, or HIV-1-Vpu^D52/56^. Infected mice were bled at 3, 7, 14, and 21-dpi. As described above, infection of hu-mice with high dose of HIV-1-WT but not HIV-1-∆Vpu resulted in high plasma viremia; at 21-dpi HIV-1-WT infected animals showed ~15-fold higher plasma viremia compared to HIV-1-∆Vpu infected animals (Figure 5A-B). Interestingly, despite its strongly attenuated BST2 antagonism phenotype in cell culture assays, HIV-1-Vpu^D52/56^ displayed a plasma viremia that was intermediate between that in WT and ∆Vpu HIV infected hu-mice; at 21-dpi, ~three-fold less HIV-1 viral particles (absolute values) were detected in plasma of hu-mice infected with HIV-1-Vpu^D52/56^ compared to HIV-1-WT infected hu-mice (Figure 5B).

Analysis of the frequency of p24^+^ T cells in spleen of infected hu-mice at 21-dpi showed that those infected with HIV-1-∆Vpu contained about ~ five-fold less p24^+^ T cells compared to those infected with HIV-1-WT as initially observed (Figure 5C). Interestingly, the frequency of p24^+^ splenic T cells in HIV-1-Vpu^D52/56^ infected hu-mice, which also showed intermediate levels of plasma viremia, was significantly lower and higher compared to HIV-1-WT and HIV-1-∆Vpu infected animals, respectively (Figure 5C). Further, type I IFN levels in peripheral blood also showed a similar trend; highest and lowest amounts of IFN were detected in plasma of HIV-1-WT or HIV-1-∆Vpu infected hu-mice, respectively, whereas intermediate amounts of IFN were detected in plasma of hu-mice infected with HIV-1-Vpu^D52/56^ (Figure 5D), thus reflecting the efficiency through which viral production and dissemination was occurring (Figure 5A and C). These data support the notion that the β-TrCP recruiting motif of Vpu is critical for optimal BST2 antagonism and for establishing efficient viremia and propagation *in vivo*.

To investigate if graded levels of plasma viremia and frequency of p24^+^ splenic T cells in hu-mice infected with Vpu mutated virus was due to differential effects of Vpu mutants on BST2 surface expression, we assessed surface levels of the restriction factor on infected T cells. As expected, compared to uninfected splenic T cells, BST2 was down regulated by 50 to 65% on p24^+^ splenic T cells from HIV-1-WT infected hu-mice, whereas as described above it was up regulated (~20%) on p24^+^ T cells from HIV-1-∆Vpu infected hu-mice (Figure 5E-F). Compared to BST2 levels on p24^-^ T cells, p24^+^ splenic T cells from HIV-1-Vpu^D52/56^ infected hu-mice showed only an average of 20-30% BST2 down regulation. That altered levels of BST2 at the cell surface correlated with the extent of BST2 antagonism and viral propagation in hu-mice infected with WT and Vpu mutant viruses is supported by the fact that p24^+^ T cells from all infected hu-mice, including those infected with Vpu mutant virus, showed comparable down regulation of CD4 (Figure 5E-F). Collectively, these data suggest that Vpu-mediated β-TrCP-dependent BST2 degradation contributes to efficient counteraction of BST2 restriction on HIV-1 release and early viral dissemination *in vivo* by ensuring that BST2 levels are efficiently reduced at the surface of infected cells.

## Discussion

The ability of Vpu to enhance HIV-1 release by counteracting the virus tethering function of BST2 has been studied extensively in culture conditions. These studies have provided important insights into the mechanisms governing Vpu-mediated antagonism of BST2 and HIV-1 release. In the present investigation we have attempted to delineate the role of Vpu in HIV-1 production and propagation during acute infection *in vivo* by infecting hu-mice with low (5000 TCID_50_) or high (500,000 TCID_50_) dose of HIV-1 expressing or not Vpu. Our data show that in condition of low dose of infection, Vpu provides a crucial “jump” in establishing early plasma viremia and HIV-1 propagation (Figure 2A). We also observed higher frequency of p24^+^ T cells in spleen of hu-mice infected with HIV-1-WT than with HIV-1-∆Vpu (Figure 2B-C). Higher plasma viremia as well as frequency of p24^+^ T cells in HIV-1-WT infected animals appeared independent of Vpu effect on CD4 or NTB-A expression as these two molecules were down regulated to a similar extent on infected cells irrespective of the presence or the absence of Vpu (Figures 2, 3 and 5). Hence, as reported by Sato and colleagues [37], it appears that down regulation of NTB-A is not sufficiently elicited by Vpu *in vivo* and the contribution of Vpu to the overall CD4 down regulation remains minor as compared to that of Nef and Env in this context. Our finding that Vpu has a minor effect on CD4 downregulation contrasts with the study of Sato and colleagues, which indeed reported a down-regulation of CD4 by Vpu in infected mice at 7 and 21 dpi, although Vpu-mediated CD4 downregulation appeared less prominent at 21dpi [37]. The reason for this discrepancy is not currently understood. It may relate to the distinct Vpu variants used in the two studies (ADA vs AD8), to the different time points at which these analyses were performed (8-10-wpi at low dose and 21dpi at high dose, in our study) or to the efficiency of infection achieved in the two studies. While a very large proportion (between 80-95%) of WT-HIV infected splenic CD4+ T cells expressed very low amounts of surface CD4 in our hu-mice (Figures 2C and 3C), Sato and colleagues detected significant CD4 expression at the cell surface of a sizeable number (~74%) of WT HIV-1-infected cells at 7-dpi, despite expression of both Vpu and Nef.

In contrast to its negligible effect on CD4 surface expression, Vpu induced a very strong down regulation of BST2; the presence of Vpu resulted in 50-75% BST2 down regulation on infected T cells, and interestingly, lack of Vpu resulted in significant increase in BST2 levels on infected T cells compared to the uninfected subset (Figure 2D). Similar to low dose infection, high dose infection of hu-mice revealed a Vpu-dependent down regulation of BST2 at the cell surface of infected cells and an initial burst of viral replication and propagation, although the difference in plasma viremia between HIV-1-WT and HIV-1-∆Vpu infected animals (~15-fold at 21-dpi) was not as significant compared to those infected with low dose at early time points (~150-fold at 4-wpi) (Figures 2 and 3). Together, these findings are consistent with the notion that Vpu-mediated BST-2 antagonism is critical for HIV-1 replication and propagation *in vivo*, especially at early times post-infection. They further suggest that BST2 restriction represents a major barrier during early infection when the predominant mode of viral transmission is likely to occur through cell-free virus. Hence, BST-2 antagonism by Vpu during this critical phase would be required to ensure the efficient initial viral expansion by cell-free virus necessary for establishing infection, particularly in the face of a host environment where a strong antiviral IFN response is triggered early on [38]. Interestingly, the fact that HIV-1 ∆Vpu replication could reach levels similar to those of HIV-1-WT at late time points post infection at low dose suggests that over time BST2 antagonism by Vpu appears less imperative most likely because viral cell-to-cell transmission, a form of viral spread that is highly efficient [39,40] and perhaps less sensitive to BST 2 restriction [9,41], becomes the predominant form of viral propagation. Thus, the reduced difference in viral outgrowth between WT and ∆Vpu HIV-infected animals at high dose despite similar effect on BST2 expression is likely to result from the faster kinetic of cell-to cell propagation that occurs under these supra physiological conditions (Figure 3).

The BST2 antagonism phenotype displayed by the mutant in the S52,56 motif of Vpu in cell culture assays was not predictive of the phenotype of this mutant *in vivo* (Figures 4 and 5), The reasons for this discrepancy remain unknown, but may involve higher expression levels of BST2 in the cellular systems used *in vitro*, such that weaker activities against BST2 are missed. Analysis of the HIV-1-Vpu^D52/56^ mutant suggests that the β-TrCP recruiting determinants of Vpu contribute to efficient counteraction of BST2 restriction on HIV-1 release and early viral dissemination *in vivo* (Figure 5). The fact that lack of β-TrCP interaction by Vpu is associated with a complete impairment of Vpu-mediated CD4 degradation further suggests that the residual initial jump of viremia and dissemination displayed by this mutant is independent of Vpu-mediated CD4 down regulation. That BST2 down regulation is critical for HIV-1 propagation is further supported by analysis of HIV-1-Vpu^D52/56^ infected hu-mice. These mice showed intermediate plasma viremia and frequency of p24^+^ T cells compared to WT or ∆Vpu infected animals (Figure 5A-C) and these correlated with a BST2 expression that was distinctly lower on p24^+^ than p24^-^ T cells (Figure 5E-F). Similarly, when the BST2 phenotype of HIV-1-∆Vpu animals is taken into consideration, down regulation seems to be almost 70 to 90% and 40-50% on p24^+^ T cells in WT or Vpu^D52/56^ infected animals, respectively, thus highlighting the ability of this mutant to still interact with BST2 and retain a partial antagonism towards the restriction factor [12,13,16]. At present, cause(s) of BST2 up regulation by HIV-1 infection in the absence of Vpu is not clear. It is possible that *in vivo* HIV-1 infection may up regulate BST2 levels in IFN dependent and independent manner and that the latter may reflect potential effects of other HIV-1 proteins such as Nef, which has been shown to up regulate BST2 levels on dendritic cells [42]. Alternatively, the upregulation of BST2 on T cells infected with Vpu-defective virus may well be related to “local” innate responses. Indeed, BST2 itself has been reported to act as an innate sensor of HIV-1 assembly so that upon restriction of Vpu-defective HIV-1 release it mediates signaling and induces NFκB-dependent proinflammatory gene expression [43]. Nevertheless, both modes of BST2 up regulation (systemic vs local) appear susceptible to Vpu-mediated antagonism.

Our data suggest that the BST2 restriction is providing most of the growth retardation of the Vpu-defective and S52,56 motif mutant since the contribution of Vpu to the overall reduction of CD4 surface levels appears negligible. However, we cannot completely exclude that the contribution of Vpu-mediated CD4 degradation to the early burst of viral propagation may be masked by Nef. Indeed, it is well known that Vpu and Nef effects on CD4 are taking place at different time during infection and are spatially separated in infected cells: Vpu promotes ER-associated protein degradation (ERAD) of newly synthesized CD4 whereas Nef stimulates endocytosis of CD4 once it has reached the plasma membrane [44]. Therefore, effects of CD4 on envelope processing and particle infectivity are also expected to be masked by Nef function, as the inhibitory effect of CD4 on Env will occur before Nef can target it. Analysis of the dynamic of infection of HIV-1 virus encoding Vpu mutants that are unable to target BST2, but yet competent for CD4 degradation, should provide complementary information about the contribution of Vpu-mediated CD4 degradation to the establishment of early HIV-1 expansion and propagation.

Lastly, our data also provide a plausible explanation for the pandemic nature of HIV-1 group M but not N. While Vpu of HIV-1 group M shows strongest BST2 antagonism with contribution from its BST2-binding TMD and β-TrCP-recruiting S52/56 motifs, some Vpu variants of group N, like the Vpu^D52/56^ mutant, are less efficient at counteracting BST2 most likely because they are impaired in their ability to recruit β-TrCP and degrade the mislocalized surface BST2 (as well as de novo synthesized CD4) [45,46].

## Conclusions

Our findings support the notion that optimal BST2 antagonism by HIV-1 Vpu is critical for efficient early viral expansion and dissemination during acute infection and as such is likely to confer HIV-1 increased transmission fitness.

## Methods

### Humanized mice

NOD-scid IL2Rγ^null^ (NSG mice) were purchased from Jackson laboratory and maintained under specific pathogen free conditions. Human cord blood was obtained from the Cord Blood Research Bank, Sainte-Justine hospital, Montreal, following approval of a research protocol by the Sainte-Justine Hospital institutional review board and written informed consent from donors. Cord blood-derived human CD34^+^ hematopoietic stem cells were isolated by positive selection using human CD34^+^ selection kit (Miltenyi Biotech). Purified cells were suspended at a concentration of 1 × 10^6^ cells per ml of complete RPMI (10% fetal bovine serum (FBS), 100U/ml of penicillin/streptomycin) and cultured for 4 hours in the presence of recombinant human cytokines: Stromal Cell Factor (SCF, 50 ng/ml), Thrombopoietin (TPO, 25 ng/ml), Flt-3 ligand (Flt-3 L, 50 ng/ml) and Interleukin-3 (IL-3, 10 ng/ml). All cytokines were obtained from PeptroTech, USA. Cultured stem cells were pelleted and suspended in Dulbecco’s Phosphate Buffered Saline (PBS) (Invitrogen, Canada) at 1 × 10^6^ stem cells per ml. Six to 7 week old NSG mice were sub-lethally X-ray irradiated (250 rads) and 24 hours later 1 × 10^5^ stem cells were injected intravenously. More than 90% of reconstituted mice showed the presence of human T cells in the blood 12 to 16 weeks post-engraftment.

### Antibodies

CD14-PE-TR, CD4-PERCP CY5.5, CD45RA-PE-CY7, CD45RO-APC, CD3-PB, CD8-APC-CY7, NTB-A-PE antibodies were obtained from Biolegend. Anti-p24, PE or FITC-coupled, were obtained from Beckman-Coulter. Goat anti-rabbit Alexa633 was from Invitrogen. Anti-GFP was from Sigma while anti-Vpu and anti-BST2 antibodies were generated in the laboratory as previously described [16].

### Cell culture

HEK 293 and 293 T cells, HeLa cells, HeLaTZM-bl cells were maintained in complete DMEM as previously described [47]. Primary CD4^+^ T cells were purified from peripheral blood mononuclear cells (PBMCs) isolated form healthy donors by negative selection kit (Stem Cell, Canada) and suspended in complete RPMI at 1 × 10^6^ cells/ml. CD4^+^ T cells were activated in the presence of PHA (4 μg/ml) and IL-2 (100U/ml) for 24 hours, centrifuged (900 rpm), and suspended in complete RPMI/IL-2 [47]. PBMCs were obtained from healthy adult donors who gave written informed consent in accordance with the Declaration of Helsinki under research protocols approved by the research ethics review board of the Institut de Recherches Cliniques de Montréal (IRCM).

### Construction of Vpu mutants

The infectious CCR5-tropic pNL4.3-Ada-GFP proviral DNA (HIV-1 WT) was derived from a pNL4.3 construct that co-expresses Nef and GFP (green fluorescent protein) from an internal ribosome entry site-containing open reading frame (ORF) (NL-GI) [48]. NL-GI was rendered CCR5-tropic by transferring the SalI-BamHI fragment from a well characterized CCR5-tropic construct Ada (NLHXADA) as described previously [49]. The resulting pNL4.3-Ada-GFP proviral construct, which encodes a Vpu protein from ADA, was used for generating Vpu mutants using standard molecular biology techniques. Vpu-deficient mutant (HIV-1-∆Vpu) was generated by subcloning a SalI-KpnI fragment from the Vpu-defective pNLVpuDEL-1 [50] into pNL4.3-Ada-GFP. VpuDEL1 contains a deletion of 48 nucleotides immediately following the initiator methionine codon of the *vpu* ORF plus a 7-nucleotide insertion which leads to a premature termination of the protein after 14 missense codons were introduced. The Vpu^D52/56^ mutant was generated as follows. In brief, 2.9 kb SalI-BamHI fragment encompassing Vpu was subcloned into pSVCMV. In this background, serine 52 and 56 residues in the CTD of Vpu were mutated to aspartic acid (Vpu^D52/56^) by Quickchange mutagenesis (Stratagene). The mutated DNAs were sequenced and cloned into the pNL4.3Ada-GFP backbone.

### Cell transfection, BST2 expression and virus release assay

Proviral DNAs expressing WT or mutated Vpu were characterized by transfecting HeLa cells. In brief, 4 μg of proviral DNA (pNL4.3-Ada-GFP-WT, pNL4.3-Ada-GFP-∆Vpu, pNL4.3-Ada-GFP-Vpu^D52/56^) was transfected into HeLa cells using lipofectamine (Invitrogen). Forty-eight hours later, cell free virus in culture supernatants was pelleted and lysed in the presence of protease inhibitors as described previously [16]. Cells were detached and an aliquot was stained for BST2 and analyzed by flow cytometry. Remainder of HeLa transfectants were lysed in the presence of protease inhibitors. An aliquot of virus and cell lysate was Western blotted for anti-p24, anti-Vpu and anti-GFP. Relative release of virus was determined by comparing ratio of Gag (p24) band signal in cell-free virus over total Gag-related band signals (virion-associated plus cell-associated Gag-related bands) in WT samples to Vpu-deleted or mutated samples (ratio in WT was treated at 100%).

### Production of virus

5 × 10^6^ 293 T cells were plated in 100 mm tissue culture plate and 24 hours later cells were transfected with 20 μg of proviral DNA by CaCl_2_ method. Culture supernatants were collected 48 hours post-transfection and virus was pelleted over a 20% sucrose cushion as described [16] The pelleted virus was titrated using HeLaTZM-bl indicator cell line for determining infectious units. For determining tissue culture infectivity dose (TCID_50_) total PBMC from healthy donors were activated in the presence of PHA (4 μg/ml in complete RPMI). Twenty four to 48 hours later, cells were washed, counted and plated at 2 × 10^5^ cells/well in a flat bottom 96-well plate and infected with serial dilution of purified virus. At 7 days post infection, supernatant was collected and p24 levels were estimated by anti-p24 ELISA and TCID_50_ was calculated according to the Spearman-Karber method.

### Primary CD4 T cell infection

Purified CD4^+^ T cells from healthy donors were PHA-activated for 24 hours and 1 × 10^6^ cells were infected with Vpu-sufficient, deficient or Vpu-mutated HIV-1 at an MOI of 1 in the presence of polybrene (8 μg/ml). Four hours later, cells were washed twice and cultured in complete RPMI containing IL-2 (100U/ml). At different time points post infection, an aliquot of cells was removed, stained for BST2 and CD4, and analyzed by flow cytometry. Culture supernatant was collected, filtered and release of cell-free virions was determined as follows. Two hundred microliters of supernatant was used in duplicate for infecting 20,000 HeLaTZM-bl cells (in 24 well plates) for 2 hours in the presence of polybrene and cultured in complete DMEM. At 48 hours post infection, HeLaTZM-bl cells were washed twice with cold PBS and lysed in luciferase buffer. Lysates were spun (13,000 rpm for 10 min at 4°C) and luciferase units were determined using the GlowMax assay (Promega).

### Infection of hu-mice

NOD-scid IL2Rγ^null^ (NSG) mice reconstituted with human CD34^+^ stem cells were infected intraperitoneally with 5,000 or 500,000 TCID_50_ of HIV-1 in 200 μl of complete RPMI. Medium was used for mock infection.

### Mouse techniques

Hu-mice were bled at different time points post infection. Plasma was collected and PBMCs were purified over Ficoll. Plasma was used for estimating HIV RNA copy numbers (plotted as log_10_ values) and type I IFN levels. PBMCs were then stained with a combination of fluorescently labeled antibodies to CD14, CD4, CD45RA, CD45RO, CD3, CD8 and BST2. For intracellular staining, surface stained cells were fixed and permeabilized (Cytofix/Cytoperm kit from BD Biosciences) and stained for HIV Gag protein. Infected hu-mice were sacrificed by cardiac puncture and spleen was harvested. Red blood cells in spleen were lysed using ACK buffer (Invitrogen) followed by surface and intracellular staining for various proteins as described for PBMCs. Flow cytometry data were collected on a CYAN flow cytometer and analyzed by Flowjo software. Mice were handled as per Canadian Committee for Animal Care guidelines and the protocol was approved by the IRCM animal care committee.

### Quantitation of HIV-1 RNA

HIV-1 viral load was determined in 100 μl of plasma. In brief, 100 μl of plasma was diluted to 1 ml with normal human serum and RNA copy numbers were determined using the Abbott real time HIV-1 M2000 kit. The detection limit of the assay is 40 copies/ml.

### IFN measurement

The assay to measure bioactive type 1 IFN levels was as described previously [47] Briefly, 5 to 10 μl of plasma obtained from hu-mice was mixed with 180 μl of human type I IFN reporter HEK-Blue cells (Invivogen; 180,000 cells/ml), incubated for 24 to 48 hours, and an aliquot of supernatant was mixed with Quantiblue (Cedarlane). Change in optical density was measured at 650 nm using a spectrophotometer. Type I IFN concentration (U/ml) was extrapolated from the linear range of a standard curve generated using known amounts of type I human IFN (PBL Interferon Source).

### Statistical analyses

Data are expressed as average with standard deviation (SD). Statistical significance between different groups of hu-mice was determined by unpaired student’s *t* test, while statistical significance between subset of cells from the same sample was determined by paired student’s *t* test (for eg., BST2 levels on p24^-^ versus p24^+^ T cells). *, **, and *** signify p ≤ 0.05, p ≤ 0.005, and p ≤ 0.0005, respectively.

## Competing interests

The authors declare that they have no competing interest.

## Authors’ contributions

VPD and EAC conceived and designed the experiments. VPD and FH performed experiments while MMD and EH generated hu-mice. VPD and EAC analyzed the data. VPD, EAC and EH wrote the paper. All authors read and approved the final manuscript.

## Supplementary Material

Additional file 1: Figure S1 Hu-mice infected with low dose of HIV-1-WT or HIV-1-ΔVpu were bled at different time points post infection. Mononuclear cells were purified on Ficoll gradient and stained with a combination of fluorescently-labeled antibodies. Frequency of p24^+^ T cells **(A)** and CD4^+^ T cells (CD3^+^CD8^-^) **(B)** in peripheral blood lymphocytes (PBL) was determined by flow cytometry analysis. Error bars represent SD; *, p ≤ 0.05. Click here for file
