# Supplementary figures and images for: Efficient BST2 antagonism by Vpu is critical for early HIV-1 dissemination in humanized mice
Source: Retrovirology. 2013 Nov 6;10:128. doi: 10.1186/1742-4690-10-128 (PMC4226203; doi:10.1186/1742-4690-10-128)

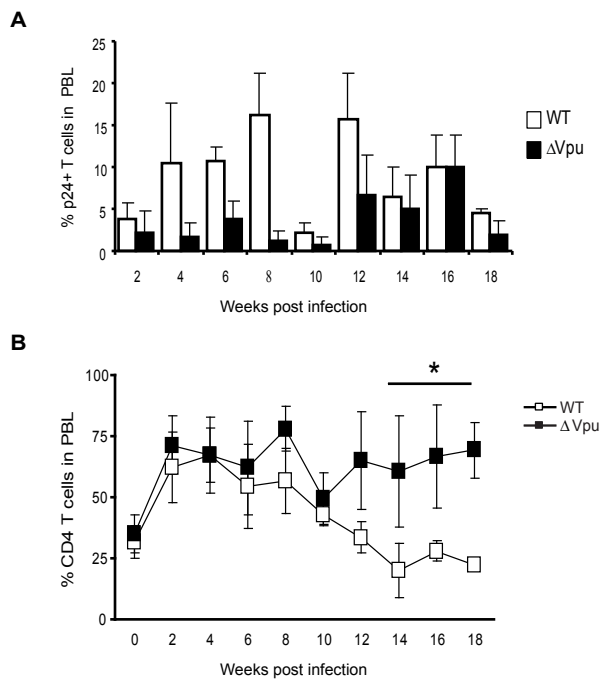

**Figure S1**

Supplement: Additional file 1: Figure S1 — Hu-mice infected with low dose of HIV-1-WT or HIV-1-ΔVpu were bled at different time points post infection. Mononuclear cells were purified on Ficoll gradient and stained with a combination of fluorescently-labeled antibodies. Frequency of p24+ T cells (A) and CD4+ T cells (CD3+CD8-) (B) in peripheral blood lymphocytes (PBL) was determined by flow cytometry analysis. Error bars represent SD; *, p ≤ 0.05. [file 1742-4690-10-128-S1.pdf]
